# Spin-coated planar Sb_2_S_3_ hybrid solar cells approaching 5% efficiency

**DOI:** 10.3762/bjnano.9.200

**Published:** 2018-08-08

**Authors:** Pascal Kaienburg, Benjamin Klingebiel, Thomas Kirchartz

**Affiliations:** 1 IEK5-Photovoltaics, Forschungszentrum Jülich, 52425 Jülich, Germany; 2 Faculty of Engineering and CENIDE, University of Duisburg-Essen, Carl-Benz-Str. 199, 47057 Duisburg, Germany

**Keywords:** antimony sulfide, hole transport material, solar cell

## Abstract

Antimony sulfide solar cells have demonstrated an efficiency exceeding 7% when assembled in an extremely thin absorber configuration deposited via chemical bath deposition. More recently, less complex, planar geometries were obtained from simple spin-coating approaches, but the device efficiency still lags behind. We compare two processing routes based on different precursors reported in the literature. By studying the film morphology, sub-bandgap absorption and solar cell performance, improved annealing procedures are found and the crystallization temperature is shown to be critical. In order to determine the optimized processing conditions, the role of the polymeric hole transport material is discussed. The efficiency of our best solar cells exceeds previous reports for each processing route, and our champion device displays one of the highest efficiencies reported for planar antimony sulfide solar cells.

## Introduction

Antimony sulfide (Sb_2_S_3_) is a promising high band gap light absorber for solar cells [1–5]. The record efficiency of 7.5% [6] is comparable to that of other less investigated materials, such as the best lead-free perovskites [7], Cu_2_O [8] and Sb_2_Se_3_ [9–10] and outperforms bismuth-halides [11–12], SnS [13] and Bi_2_S_3_ [14–16]. However, the efficiency of Sb_2_S_3_ trails behind the more thoroughly studied material systems such as lead-based perovskites [17], organic solar cells [18–19] or PbS [20], thus further technological investigation is needed. Two basic factors that impact the solar cell performance of a given material are the device architecture, which defines the mechanism of charge separation, and the deposition method for the absorber, which affects the film and electronic material quality.

**Figure 1:**
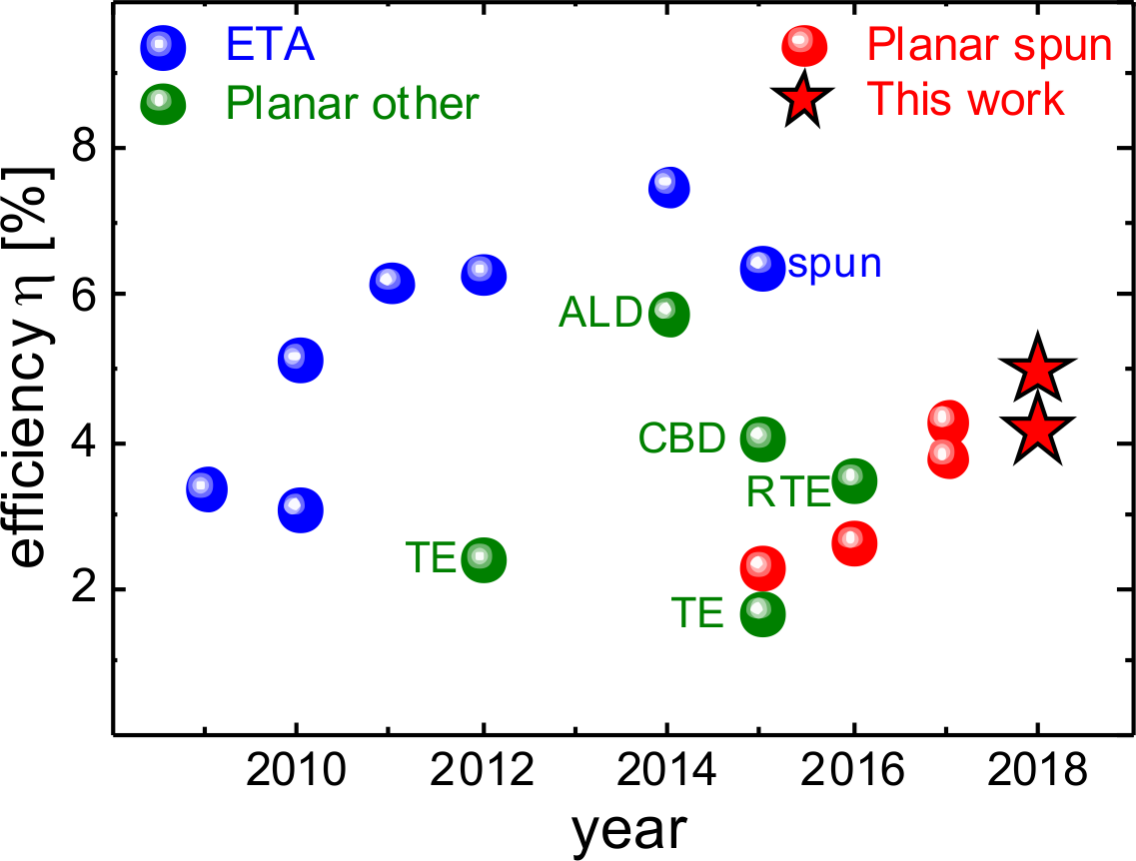
Development of Sb_2_S_3_ technology. Solar cells with extremely thin absorber architecture [1–6,29] reach the highest efficiencies. Planar devices have been produced via various methods such as atomic layer deposition (ALD) [32], chemical bath deposition (CBD) [27] and (rapid) thermal evaporation (R)TE [33–35]. As the latest development, spin-coated planar solar cells [31,36–38] reached an efficiencies >4%. This work extends the progress to almost 5%.

Sb_2_S_3_ is commonly applied in an extremely thin absorber (ETA) architecture, which is similar to that of dye-sensitized solar cells [21]. A thin absorber layer of around 10 nm [22] is deposited on a mesoporous TiO_2_ scaffold and the pores are subsequently filled with a hole transport material (HTM). Progress in terms of device efficiency can be attributed to more feasible HTMs [1–5] and improved properties of the Sb_2_S_3_ itself [6], which lead to a record efficiency of >7% as shown in Figure 1. The core idea behind the ETA concept is that thin inorganic absorber layers relax the requirements for electronic material quality [23–25] since charges are quickly extracted from the absorber. However, the benefits are restricted by recombination via trap-assisted tunneling [24] which must be compensated by increasing the absorber thickness for optimized performance. A major challenge in the processing of ETA solar cells is the improper infiltration of pores [22], which can give way to incomplete coverage of the TiO_2_ scaffold and an interface between TiO_2_ and HTM. From a conceptual perspective, planar geometries reduce the large interface area required in ETA cells for appreciable photocurrent generation and should better prevent direct contact between the electron and hole transport layers both of which should reduce recombination [26]. Indeed, despite the generally lower efficiencies, as depicted in Figure 1, planar geometries have reached slightly higher open-circuit voltages [27–28] than the best performing ETA cells [1,6] – especially when devices are compared that apply the same HTM and Sb_2_S_3_ deposition method [26,29–31].

Typically Sb_2_S_3_ is fabricated via chemical bath deposition (CBD) [2,39–41] with the drawback of a complex growth mechanism that includes heterogeneous nucleation and exponential growth which requires the precise control of processing conditions and eventually limits the process’ reproducibility [29,32]. During chemical reactions in the water bath, various antimony oxides, hydroxides and sulfates form [6,42–44] which could be detrimental to device performance. With respect to oxide formation a short air exposure was shown to increase device performance but longer exposure times deteriorated the solar cell [45]. In another study post-sulfurization reduced the Sb_2_O_3_ content and thereby the concentration of deep traps which improved solar cell performance [6].

As an alternative to CBD, spin-coating of different antimony- and sulfur-containing precursors was proposed [29,36–37]. A metal-organic complex is formed in solution which is then spin-coated and afterwards thermally decomposed. Just like for CBD [2,41] or ALD [22,32] the resulting amorphous film needs to be annealed at elevated temperatures to obtain crystalline Sb_2_S_3_. Choi and Il Seok reported an antimony–thiourea (Sb–TU) complex and demonstrated efficiencies above 5% in an ETA configuration with poly[2,6-(4,4-bis-(2-ethylhexyl)-4*H*-cyclopenta[2,1-b;3,4-b’]-dithiophene)-*alt*-4,7-(2,1,3-benzothiadiazole)] PCPDTBT as the hole transport material and efficiencies above 6% when an organic bulk heterojunction was used instead of the pure polymer [29]. Gil et al. [38] applied the same precursor to a planar device configuration and found a strong correlation between TU content and film morphology. The best morphology and device efficiency of 2.7% was obtained for Sb/TU ratios that imply a strongly sulfur-deficient stoichiometry according to the previously mentioned study [29]. However, Gil et al. [38] performed crystallization in an H_2_S atmosphere which could increase the sulfur content in the film. In a follow up work, Sung et al. [31] showed that rough substrates are beneficial for the formation of compact Sb_2_S_3_ films which relaxed the constraint that a good morphology requires low TU content. Molar ratios closer to stoichiometric conditions yielded relatively compact layers and enabled higher efficiencies up to 3.8%. Although the conditions to reach a good morphology were to some extent de-coupled from the film’s chemical composition, voids can still be identified in the presented SEM images, which leaves room for further process improvement. Based on an antimony-butyldithiocarbamate (Sb-BDC) complex, Wang et al. [37] fabricated pinhole-free layers with large grains and the so-far highest reported efficiency of 4.3% for spin-coated planar Sb_2_S_3_ solar cells as can be seen from Figure 1. However, this route includes Sb_2_O_3_ as a precursor whose detrimental impact has been discussed above. While these initial results on spin-coated planar antimony sulfide solar cells are promising, many process parameters have not yet been discussed properly.

In this work, we follow the two depicted fabrication routes for spin-coated planar Sb_2_S_3_ solar cells based on different precursors [29,37]. For the Sb-TU precursor we introduce a slow annealing process that considerably improves substrate coverage. We compare both process routes in terms of morphology, electronic defects and device performance with a focus on the crystallization step. For optimized annealing conditions, we vary the hole transport material and illustrate a qualitatively different impact on device performance for the two precursor process routes. For both precursor routes, the efficiency of the presented optimized devices exceeds that of previous reports.

## Results and Discussion

In the process described in [29] antimony chloride SbCl_3_ and thiourea SC(NH_2_)_2_, or short TU, are used to form an antimony-thiourea complex [Sb(TU)_2_]Cl_3_ in the high boiling point solvent *N*,*N*-dimethylformamide DMF. While [31,38] chose 2-methoxyethanol instead of DMF as the solvent, we stuck to the original recipe with DMF. The second process route applied in this work and described in [37] uses antimony oxide Sb_2_O_3_ and butyldithiocarbamic acid BDCA, formed by reacting *n*-butylamine with CS_2_, to obtain an antimony-butyldithiocarbamate complex Sb(S_2_CNHC_4_H_9_)_3_ which is dissolved in ethanol. With reference to the formed Sb-complex and as indicated in the introduction, we will refer to the first process as Sb-TU route, and to the second process as Sb-BDC route. For both processes the spin-coated Sb-complex is thermally decomposed at around 200 °C leaving an amorphous film and then crystallized at higher temperatures in an inert atmosphere [29,37]. Details of the fabrication can be found in the Experimental section. It is noteworthy that both processes use an excess of the sulfur precursors. For the case of Sb-TU it was shown that stoichiometric crystalline Sb_2_S_3_ with an S/Sb ratio of 3/2 = 1.5 in the resulting film, which showed the best performance in an ETA solar cell, requires this initial excess of sulfur in the precursor (SbCl_3_/TU = 1.8) [29].

**Figure 2:**
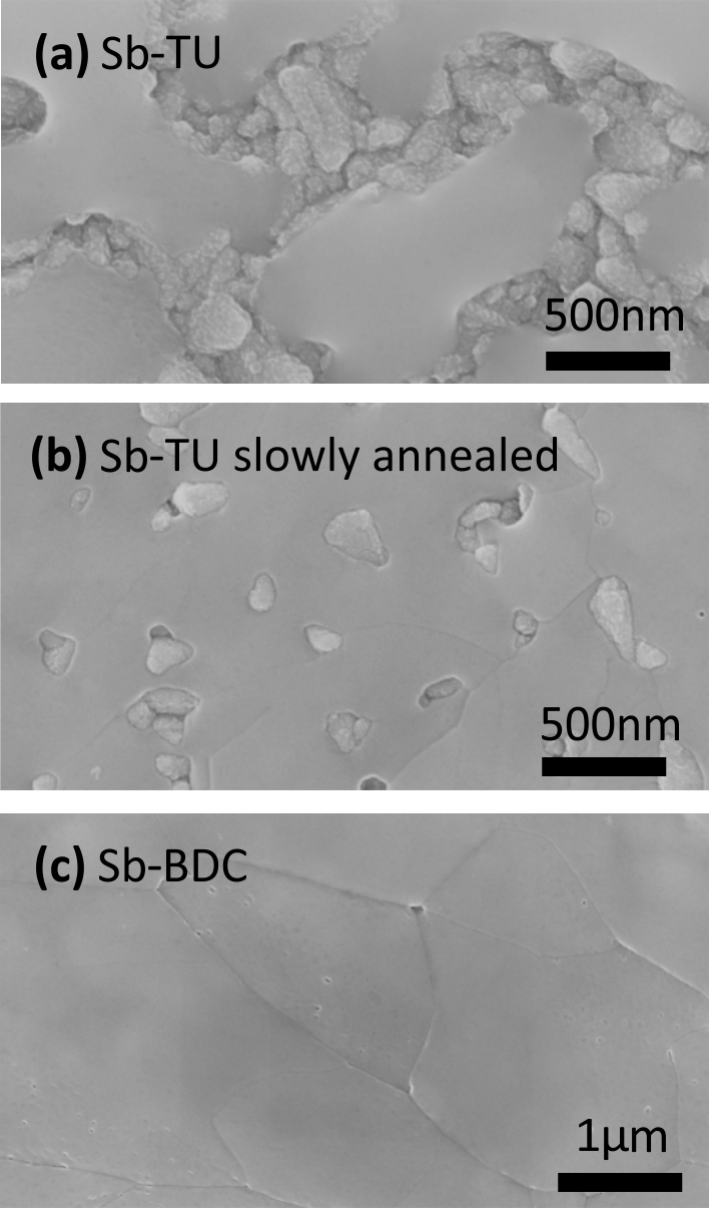
SEM images of Sb_2_S_3_ thin films after crystallization at 265 °C. Direct thermal decomposition of the spin-coated Sb-TU precursor solution leaves the substrate largely uncovered (a). An intermediate slow annealing step at 100 °C for 60 minutes reduces the pinhole area (b). The Sb-BDC based process leads to a compact layer with large grains and without pinholes (c).

In a first step, the film morphology was studied. While the Sb-TU process allows the homogeneous deposition of Sb_2_S_3_ in a mesoporous TiO_2_ scaffold which enables device efficiencies comparable to other deposition methods [29], direct thermal decomposition of the spin-coated solution at 180 °C leaves large parts of the planar substrate uncovered as can be seen in the scanning electron microscope (SEM) image in Figure 2a. Between smooth-looking domains of Sb_2_S_3_, the grains of the FTO (Pilkington TEC7) covered with spray-coated TiO_2_ are clearly visible. For better comparison, SEM images of substrates without Sb_2_S_3_ can be found in Figure S1, Supporting Information File 1. The morphology is improved by annealing the films at 100 °C directly after spin-coating for approximately 60 minutes prior to thermal decomposition at 180 °C. Figure 2b shows that this slow annealing step drastically reduces the area of pinholes in the film. Both images in Figure 2a and 2b are taken after crystallization at 265 °C. The holes are already present in the amorphous films as can be seen in the corresponding images shown in Figure S1, Supporting Information File 1. The existence of pinholes is thus not caused by crystallizing the film which emphasizes that the detailed procedure of thermal decomposition is crucial for the film morphology. Grain sizes are on the order of 500 nm. While even the optimized annealing procedure cannot fully avoid the presence of pinholes for the Sb-TU process, the Sb-BDC process leads to compact layers largely free of pinholes as shown in Figure 2c. Except for a lower crystallization temperature as will be discussed later, we closely followed the recipe reported in [37] and obtain a very similar film morphology, with grain sizes exceeding 1 μm but slightly smaller than the reported average of 6 µm. Note that the amorphous film has a porous structure as can be seen in Figure S1c, Supporting Information File 1. The compact and pinhole-free morphology is a result of crystallization during the Sb-BDC process which again underlines the apparently different mechanisms governing film formation in the Sb-BDC and Sb-TU processes.

Most deposition methods such as chemical bath deposition [2,41], atomic layer deposition [22,32], thermal evaporation [33,35,46] and the here-discussed spin-coating [29,37] produce amorphous films that are subsequently crystallized at temperatures above the minimum crystallization temperature of 250 °C for antimony sulfide [47–49]. The typical crystallization temperature is 300 °C [2,6,29,31,37] while values of 330 °C [4–5,32,50] and up to 400 °C [51] are reported. To gain insights into the crystallization behavior in terms of morphology, creation of defects and solar cell performance we produced samples crystallized on a hot plate at various temperatures in a nitrogen atmosphere. The hot plate temperature and homogeneity was confirmed by contact thermometer measurements. The lowest temperature was chosen to be 265 °C slightly above the minimum crystallization temperature of Sb_2_S_3_. The morphology was studied with SEM and atomic force microscopy (AFM). Possible changes in electronic quality with crystallization temperature were investigated via photothermal deflection spectroscopy (PDS) where the absorption coefficient of a thin-film is measured over several orders of magnitude [52–53] which cannot be achieved by standard transmission–reflection measurements using an UV–vis photospectrometer. The large dynamic range of PDS makes it a powerful tool for to study the density of states in the sub-bandgap region [53–56] including band tails that yield the Urbach energy as a measure of disorder as well as the detection of (optically active) defects in the band gap which can act as recombination centers in a solar cell.

**Figure 3:**
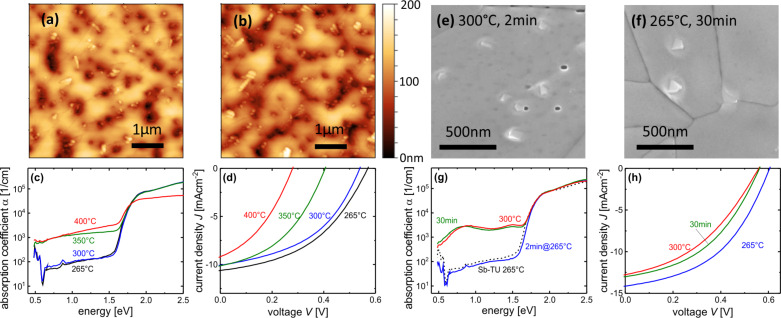
Crystallization in the Sb-TU (a–d) and Sb-BDC (e–h) process. AFM measurements of the Sb-TU route show a coarsening of the morphology at 300 °C (b) compared to 265 °C (a). Electronic defects detected with PDS (c) are created in the band gap for temperatures of 350 °C or above. Corresponding device performance (d). SEM images of the Sb-BDC process for higher crystallization temperatures (e) and longer crystallization times (f) compared to the standard (265 °C, 2 min). New features in the morphology come along with increased defect absorption (g) and deteriorated device performance (h) for conditions uncritical in the Sb-TU process.

The Sb-TU process shows a slight increase in uncovered substrate area for a crystallization temperature of 300 °C (Figure 3b) compared to a crystallization temperature of 265 °C (Figure 3a). This trend continues for higher crystallization temperatures as can be seen from AFM scans shown in Figure S2 where the corresponding SEM measurements are also presented. At 400 °C the film seems to disintegrate and macroscopic holes form. The small, bright, tapered features that are observed in the domains not covered by Sb_2_S_3_ can be attributed to the peaks of large FTO grains as can be seen from the comparison with the SEM images of the FTO in Figure S1, Supporting Information File 1. In the relevant temperature range of 265 °C to 300 °C, the Sb_2_S_3_ domains tend towards a droplet-like morphology with increasing temperature which can be interpreted as an on-going de-wetting of the substrate. The issue of de-wetting was reported for the transition from the amorphous to the crystalline phase of Sb_2_S_3_ for a thin layer on a mesoporous TiO_2_ scaffold [22,57]. For PDS measurements of the Sb-TU process, a TiO_2_ layer was spray-coated onto the glass before depositing the Sb_2_S_3_ layer because non-optimal adhesion prevents direct coating of glass with Sb-TU solution. The deposition conditions thus closely resemble those of the fabricated solar cells. The results for different crystallization temperatures in the Sb-TU process are shown in Figure 3c. For the sample crystallized at 265 °C and 300 °C, the measurement signal quickly saturates at energies above the band gap. At low energies the absorption strength of the substrate becomes comparable to that of the Sb_2_S_3_ film which is evident from the characteristic H_2_O absorption peak of Corning glass below 1 eV. While the layer crystallized at 265 °C and 300 °C behave almost identical, the defect absorption of the layer crystallized at 350 °C is increased drastically. The same holds true for the 400 °C sample. Due to the macroscopic holes in the film described earlier, the incoming light is still transmitted and not fully absorbed even at high energies which inhibits a proper analysis of the absorption coefficient. Therefore the 400 °C sample does not coincide with the other samples that match very well for energies above the band gap. The increased defect absorption for the crystallization at 350 °C or higher hints towards a lower cell performance because a higher defect density would cause increased recombination and eventually a lower open-circuit voltage *V*_oc_. To confirm this hypothesis we produced solar cells in a standard configuration described in the experimental section with P3HT as the hole transport material. The results are shown in Figure 3d and Table S1, Supporting Information File 1 and the *J*_sc_ values obtained from solar simulator measurements are confirmed by external quantum efficiency (EQE) measurements shown in Figure S4, Supporting Information File 1. The cell from an Sb_2_S_3_ layer crystallized at 300 °C shows a lower *V*_oc_ and *J*_sc_ than the cell crystallized at 265 °C. At 350 °C, the *V*_oc_ drops drastically which is consistent with the increased defect absorption observed with PDS (Figure 3c). When the film morphology degenerates at 400 °C the cell performance decays further.

Next, a similar study was conducted for the Sb-BDC process with a focus on crystallization at 265 °C and 300 °C. The SEM image in Figure 3e of the layer crystallized at 300 °C shows a similar morphology as the one crystallized at 265 °C shown in Figure 2c except that small pyramidal structures appear on top of the grains. A sample crystallized at 265 °C for 30 minutes shown in Figure 3f instead of the standard 2 minutes shows similar structures (also see the zoomed-out SEM images in Figure S3, Supporting Information File 1). The nature of these features remains unclear. Similar structures were reported for chemical-bath-deposited and evaporated Sb_2_S_3_ [27,35,47,58] and seem to be present in [37] as well. The absorption coefficient of the Sb-BDC sample crystallized at 265 °C shown in Figure 3g is almost identical to that of the Sb-TU sample crystallized at the same temperature shown in Figure 3c and drawn again in Figure 3g for direct comparison. However, the defect absorption of Sb_2_S_3_ from the Sb-BDC route crystallized at 300 °C is strongly enhanced. A sample prepared at 265 °C but with a crystallization time of 30 minutes reveals a similar increase in defect absorption. For both samples with increased defect absorption the absorption behaves non-monotonous with energy. The maxima and minima can be most-likely attributed to interference in the smooth films – which did not fully cancel out during data analysis – instead of actual variations in the materials’ density of states in the sub-bandgap region. The negative impact of the increased defect density on device performance is confirmed by comparing solar cells crystallized at 265 °C for 2 minutes and 30 minutes and at 300 °C for 2 minutes in Figure 3h and Table S2, Supporting Information File 1.

In summary, for both investigated process routes an optimized crystallization temperature of 265 °C was found which is lower than the commonly applied treatment at 300 °C. For the Sb-TU process, higher crystallization temperatures cause a de-wetting of the substrate. New features arise on top of the Sb_2_S_3_ film in the Sb-BDC process for longer crystallization times and crystallization at 300 °C. At the same time significantly increased defect formation was observed. The same conditions were uncritical in terms of defect formation for the Sb-TU process where similar degradation started only at crystallization temperatures of 350 °C. Possibly, the stoichiometry of Sb and S changes in different ways for the two process routes during crystallization. Residues from precursors used in the Sb-BDC process such as Sb_2_O_3_ whose negative impact on device performance was shown [6,45] might also lead to defect formation during crystallization. Further insights would require a correlation between electronic defect creation at higher crystallization temperatures with changes in the chemical composition and microstructure of the Sb_2_S_3_ layer.

**Figure 4:**
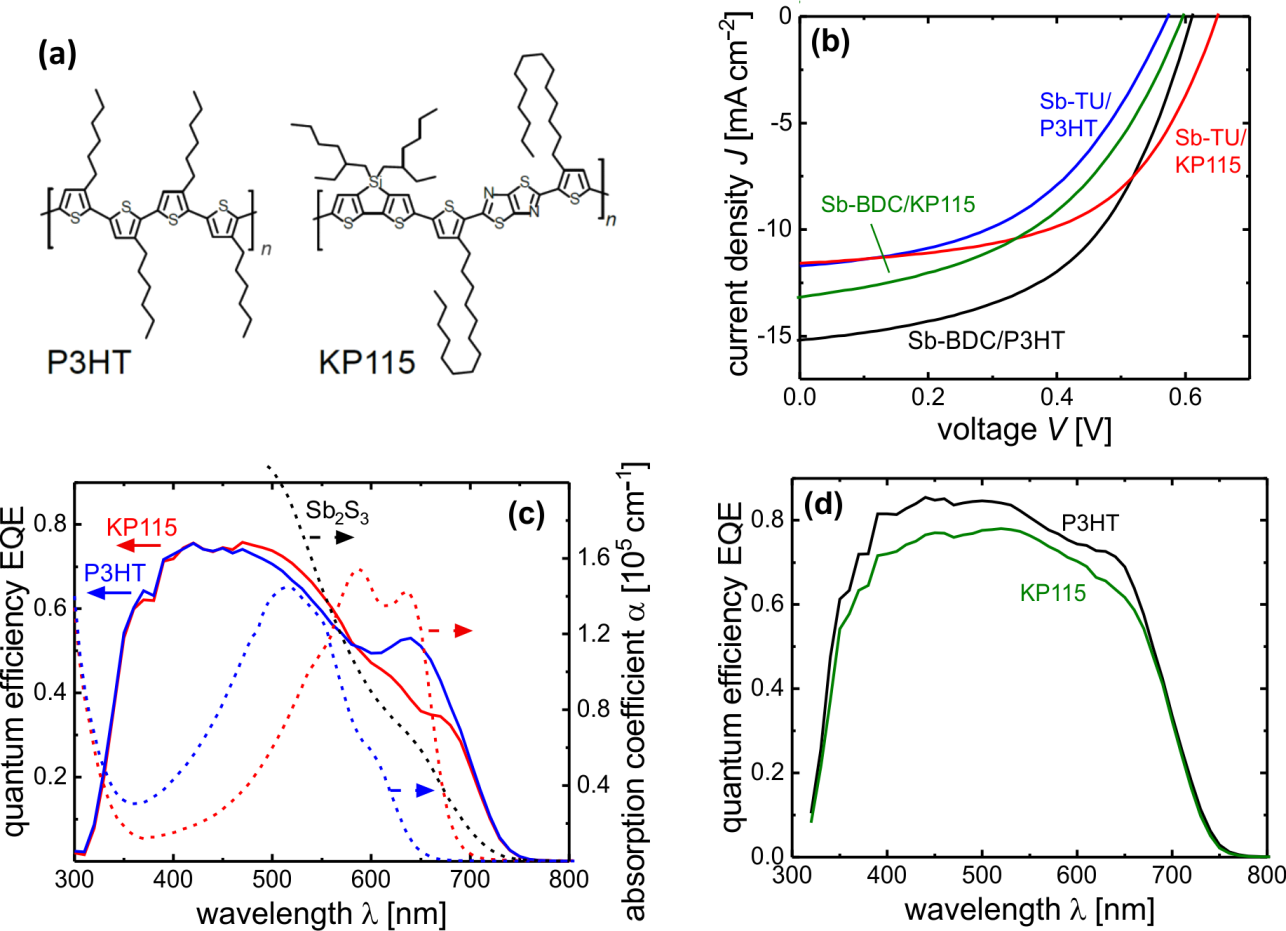
Chemical structure of the applied polymers (a). Sun simulator (b) and external quantum efficiency (c,d) measurements of FTO/TiO_2_/Sb_2_S_3_/HTM/MoO*_x_*/Ag samples. The EQE for Sb-TU samples are shown in (c) together with absorption coefficients of Sb_2_S_3_ and the two HTMs which absorb parasitically. EQE for Sb-BDC samples are shown in (d).

With a focus on the two different hole transport materials P3HT and KP115 shown in Figure 4a, the *J*–*V* curves and solar cell performance of the best devices for the two process routes are compared in Figure 4b and Table 1. The Sb-BDC process reaches higher device efficiencies compared to the Sb-TU process, mostly due to the approximately 30% higher *J*_sc_. EQE measurements shown in Figure 4c and 4d confirm this result, which can be explained by a thicker Sb_2_S_3_ layer of 190 nm compared to 100 nm for the Sb-TU process. Since Sb_2_S_3_ absorbs up to longer wavelengths than both polymers as can be seen from Figure 4c, the falling edge of the EQE can be attributed to Sb_2_S_3_ absorption. The inflection point of the EQE [59] yields a band gap of 1.79 eV. Although fill factor (FF) and *V*_oc_ do not vary as much between the processes as the *J*_sc_, it is noteworthy that despite a lower efficiency the highest *V*_oc_ of 650 mV is obtained for the Sb-TU process with KP115 as HTM. One possible reason would be a lower recombination rate due to fewer deep defects in the Sb-TU process. The shunt behavior caused by the pinholes in the Sb-TU process depends on the choice of HTM which could limit the *V*_oc_ in the case of P3HT. This explanation is consistent with the negligible *V*_oc_ difference between different HTMs in the case of the pinhole-free layers obtained from the Sb-BDC process.

**Table 1:** Device performance of samples shown in Figure 4. The efficiency for EQE-corrected *J*_sc_ is given in the last column.

Process	HTM	*V*_oc_ [mV]	FF [%]	*J*_sc_ [mA cm^−2^]	PCE [%]	*J*_sc,EQE_ [mA cm^−2^]	PCE_corr_ [%]

Sb-TU	P3HT	573	47.8	11.7	3.20	12.1	3.31
Sb-TU	KP115	650	54.8	11.6	4.13	11.7	4.16
Sb-BDC	P3HT	611	52.1	15.2	4.83	15.6	4.97
Sb-BDC	KP115	596	46.6	13.2	3.65	14.4	3.98
Sb-BDC	P3HT^a^	597	49.0	13.9	4.06	14.4	4.22

^a^Fabricated on same day as Sb-BDC/KP115 device for direct comparison, graph shown in Figure S5, Supporting Information File 1.

The two semiconducting polymers poly(3-hexylthiophene) P3HT and poly[(4,4′-bis(2-ethylhexyl)dithieno[3,2-*b*:2′,3′-*d*]silole)-2,6-diyl-*alt*-(2,5-bis(3-tetradecylthiophen-2-yl)thiazolo[5,4-*d*]thiazole)-2,5-diyl] KP115 depicted in Figure 4a allow high efficiencies at comparably thick layers when applied as absorbers in organic solar cells due to good transport properties [60–62]. Photons that are absorbed by the polymer generate excitons that can only diffuse up to around 10 nm before they recombine. While a photocurrent contribution from the polymer was demonstrated for certain Sb_2_S_3_ ETA cells [29] where the interface area is large and close-by, absorption in planar Sb_2_S_3_ cells by the polymer is to a large extent parasitic [27]. The applied polymers differ in band gap as can be seen from the measured absorption spectra in Figure 4c and the position of the highest occupied molecular orbital (HOMO). The HOMO of Sb_2_S_3_ obtained from ultraviolet photon spectroscopy (UPS) measurements is reported to lie between 5.3 and 5.5 eV [30,51,63] with one report claiming 5.9 eV [32]. Reported HOMO values of the polymers are 4.9–5.1 eV for P3HT [64–65] and 5.3–5.4 eV for KP115 [61,66] and are obtained from cyclic voltammetry measurements, which yields lower lying HOMO levels than UPS measurements [67]. The HOMO of KP115 is thus better aligned with the valence band of Sb_2_S_3_ which could be beneficial for the *V*_oc_ and FF. Indeed, for the Sb-TU process both values are significantly larger for the better-matching KP115. However, this is not the case for the Sb-BDC process where the Sb_2_S_3_/KP115 cell even shows a slightly lower *V*_oc_ and FF – also when compared to an Sb_2_S_3_/P3HT cell prepared on the same day as listed in Table 1 and shown in Figure S5, Supporting Information File 1. For the thinner Sb_2_S_3_ layers from the Sb-TU process the shape of the EQE spectra in Figure 4c differs significantly between the Sb_2_S_3_/P3HT and Sb_2_S_3_/KP115 cells for wavelengths above 500 nm. This can be explained by the different absorption spectra which are plotted together with the EQE data. P3HT has a larger band gap than KP115 so that P3HT does not absorb above approximately 650 nm. The incident light is instead transmitted to the metal back contact where it is reflected back through the P3HT into the Sb_2_S_3_ absorber where it contributes to the photocurrent. The decreased parasitic absorption above 650 nm causes a maximum in the EQE at roughly that wavelength. KP115 on the other hand still absorbs at 650 nm so that the EQE of the Sb_2_S_3_/KP115 device is decreased almost up to 700 nm. Since organic polymers have narrow absorption bands, the higher band gap P3HT continues to absorb parasitically further in the blue wavelengths than KP115. This could explain the enhanced EQE for the Sb_2_S_3_/KP115 device at wavelengths below 550 nm where the enhancement is however less pronounced because more light is already absorbed in the Sb_2_S_3_ than at longer wavelengths. Consequently, the *J*_sc_ of the Sb_2_S_3_/P3HT is 4% higher than that of Sb_2_S_3_/KP115. For the Sb-BDC process similar but less pronounced EQE features are observed as can be seen for the samples prepared on the same day and shown in Figure S5, Supporting Information File 1. One reason is that the HTM layer could be coated thinner (30 nm compared to 50 nm in the Sb-TU process) since the Sb-BDC route results in smoother and pinhole-free layers whereby the amount of parasitic absorption is decreased. Another reason is that the Sb-BDC process yields thicker Sb_2_S_3_ layers (190 nm vs 100 nm from the Sb-TU process) so that more light is absorbed in the Sb_2_S_3_ before it reaches the HTM.

In summary, the investigated Sb_2_S_3_ process routes do not seem to be generally limited by an unmatched HOMO of the HTM. For the Sb-TU route, the best results are obtained with KP115, but for the Sb-BDC route, P3HT performs best. The positive effects for the films with pinholes from Sb-TU might be attributed to a better shunt blocking in the case of KP115, which is currently under further investigation. Another reason could be that the Sb_2_S_3_/HTM interface is limiting for the Sb-TU process, whereas the bulk of Sb_2_S_3_ becomes limiting for the thicker absorber layers from the Sb-BDC. The EQE spectra and corresponding *J*_sc_ values are clearly influenced by the absorption spectra of the applied polymer. Parasitic absorption is more pronounced for polymers with a band gap closer to that of Sb_2_S_3_, for thinner layers of Sb_2_S_3_ and for thicker polymer layers.

## Conclusion and Outlook

We compared two spin-coating processes based on different precursors for Sb_2_S_3_ solar cells in a planar configuration. For both fabrication routes, an optimum crystallization temperature of 265 °C – slightly above the minimum crystallization temperature and lower than the typically reported 300 °C – was found. Depending on the process, the exact heating procedure with regard to intermediate temperature annealing or crystallization time is critical for morphology, defect density and device performance. The best choice of hole transport material depends on the precursor route and is likely related to whether pinholes are present or not. Optimized process parameters for both processing routes enabled increased device efficiencies with respect to the corresponding literature reports. The Sb-BDC process with P3HT as HTM marks one of the highest efficiencies for planar Sb_2_S_3_ solar cells and is only outdone by fabrication via cumbersome atomic layer deposition [32] as can be seen from Figure 1.

**Figure 5:**
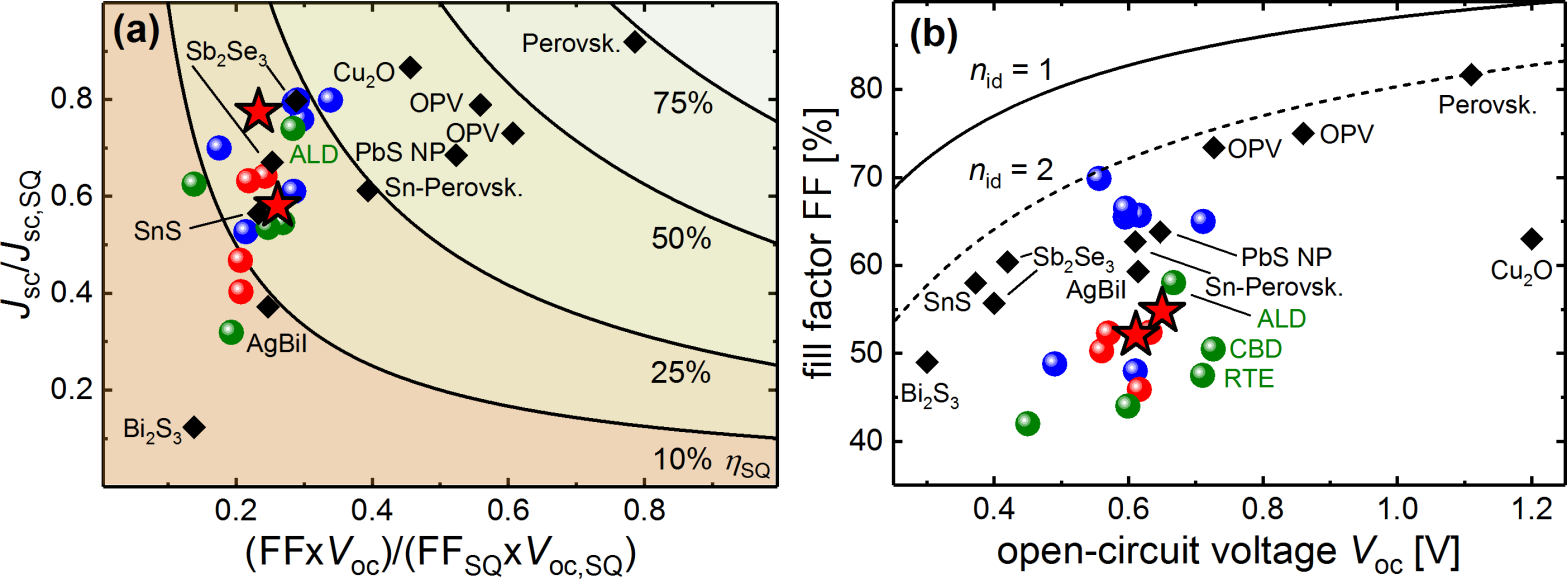
Limitations of different Sb_2_S_3_ technologies (same data and color code as in Figure 1) and other absorber materials [7–10,12–13,16–20]. Experimental solar cell performance compared to the Shockley–Queisser SQ limit (a). FF data compared to the maximum obtainable FF for given experimental *V*_oc_ (b).

In analogy to [68] the losses relative to the Shockley–Queisser limit [69] in *J*_sc_ and FF-*V*_oc_ product is shown in Figure 5a. This work, as well as other reports on Sb_2_S_3_ solar cells, reaches relatively high *J*_sc_ values when compared to some less-established absorber materials mentioned in the Introduction. Electronic losses in FF and *V*_oc_ are more severe. Figure 5b further deconvolutes the critical parameters by comparing the FFs of the same solar cells to the theoretical maximum FF [70–71] which is a function of the *V*_oc_ and the ideality factor *n*_id_. The Shockley–Queisser limit of the *V*_oc_ is almost 1.5 V for Sb_2_S_3_ so that *V*_oc_ losses clearly exceed losses in the FF which are nevertheless noticeable for all planar Sb_2_S_3_ solar cells as can be seen from Figure 5b. The high *J*_sc_ of Sb_2_S_3_ underlines the general suitability as an absorber but further improvements must tackle the deficient fill factor and especially the low open-circuit voltages obtained for all Sb_2_S_3_ solar cells up-to-date.

Regarding future technological progress, our study shows that for otherwise similarly processed samples the choice of precursor is crucial for the resulting film morphology and device efficiency. Complex chemistry offers versatile options for the design of new precursors that could further enhance the efficiency of solution processed Sb_2_S_3_. For any new process pinhole-free layers with tuneable thickness and control over stoichiometry are desirable and correlation to device performance is insightful. Post-processing, such as sulfurization from gas [32] or liquid [6] sulfur sources might reduce the density of electronic defects in Sb_2_S_3_ for certain deposition methods that leave sulfur deficient films [29,72]. Another aim in terms of fabrication is to exploit the anisotropic nature of Sb_2_S_3_ by aligning the 1D ribbons with the direction of charge transport between the contacts. A beneficial effect was demonstrated for the structurally identical Sb_2_Se_3_ [9]. A proper substrate choice or embedding a seed layer might be the key to directed growth in Sb_2_S_3_.

## Experimental

**Chemicals:** All chemicals were purchased from Sigma-Aldrich except for KP115 which was purchased from 1-material. Dried solvents with analytical (p.a.) quality were used.

**Sb_2_S_3_ layers from Sb-TU precursor:** 1 mmol of SbCl_3_ was dissolved in 1 mL of DMF and stirred for 30 min. The solution was then added to TU with an SbCl_3_/TU molar ratio of 1:1.8, stirred again overnight and filtered before use. The solution was spin-coated at 70 rps for 40 s with 10 s of acceleration. The samples were then annealed for 60 min at 100 °C on a hot plate which was then heated up to 180 °C where the samples remained for another 10 min followed by crystallization on another hot plate for 30 min at the temperature indicated in the text with a standard crystallization temperature of 265 °C. We chose 265 °C slightly above the minimum crystallization temperature of Sb_2_S_3_ as the lowest temperature to account for minor temperature fluctuations across the hot plate and differences between the hot plate’s surface temperature and the film temperature. Because of the inhomogeneous morphology of the Sb-TU process no single layer thickness can be given but an average thickness of 100 nm was determined from AFM measurements on glass/TiO_2_/Sb_2_S_3_. Layers for SEM and AFM imaging were prepared on FTO TEC7 by Pilkington after spray-coating of TiO_2_. Layers for PDS and UV-Vis measurements were coated on Corning glass after spray-coating of TiO_2_. Sb_2_S_3_ was exclusively processed under an inert N_2_-atmosphere.

**Sb_2_S_3_ layers from Sb-BDC precursor:** 1 mmol of Sb_2_O_3_ was dissolved in a solution of 1.5 mL CS_2_ mixed with 2 mL ethanol and stirred for 1 h. 2 mL *n*-butylamine were then added in a dropwise manner and the solution was allowed to cool down four times during the preparation since the formation reaction is exothermal and CS_2_ has a boiling point of 46 °C. The solution was stirred again overnight and filtered before use. The solution was spin-coated at 133 rps for 30 s with 3 s of acceleration. The samples were then annealed for 1 min at 200 °C on a hot plate and then crystallized on another hot plate as indicated in the text with a standard crystallization temperature of 265 °C for 2 min. The resulting layer thickness is 190 nm. Layers for SEM and AFM imaging were prepared on FTO TEC7 substrates by Pilkington after spray-coating of TiO_2_. Layers for PDS and UV–vis measurements were coated directly on Corning glass since – in contrast to the Sb-TU route – adhesion on glass was uncritical. Sb_2_S_3_ was exclusively processed under an inert N_2_-atmosphere.

**Solar cells:*** N*-*i*-*p* stack of FTO/TiO_2_/Sb_2_S_3_/HTM/MoO*_x_*/Ag. FTO TEC7 substrates by Pilkington were structured by Kintec. A compact layer of TiO_2_ was obtained by spray-coating a 0.2 M solution of titanium diisopropoxide bis(acetylacetonate) 75 wt % in ethanol on a hot plate at roughly 470 °C. After cooling down to about 200 °C, samples were transferred to a glovebox with N_2_ where they were further processed on the next day. Sb_2_S_3_ layers were fabricated as described above. P3HT was dissolved in chlorobenzene (CB) and stirred overnight at 65 °C. The cooled solution was spin-coated at 6000 rpm for 120 s with an acceleration time of 3 s after which the samples were annealed at 130 °C for 15 min. For Sb-TU samples a 15 mg/mL solution was used and for Sb-BDC samples a 10 mg/mL solution was used. KP115 was dissolved in 1,2-dichlorobenzene (DCB) with a concentration of 10 mg/mL and stirred overnight at 110 °C. The hot solution was spin-coated at for 120 s with an acceleration time of 3 s after which the samples were dried in a closed petri dish for 3 h. For Sb-TU samples the spin speed was 1500 rpm and for Sb-BDC samples 4000 rpm. Resulting polymer thicknesses were measured on a glass reference and adjusted to 50 nm for Sb-TU samples and 30 nm for Sb-BDC samples. The different optimized polymer layer thickness is due to the rougher and pinhole-containing Sb-TU films. Finally 30 nm of MoO*_x_* and 200 nm of Ag were thermally evaporated. The metal contact configuration where MoO*_x_* forms a tunneling junction with the HTM is typical for organic solar cells in an *n*-*i*-*p* configuration. The cell area was 0.16 cm^2^.

**Layer and device characterization:** The scanning electron microscope was a Zeiss (Leo) Gemini 1550 with Shottky field-emission cathode and an in-lens detector. The lateral resolution equals approximately 1 nm at 20 kV and the measurement was performed under a vacuum base pressure of 10^−6^ mbar. UV–vis measurements were performed with a Lambda 950 spectrophotometer from PerkinElmer equipped with an integrating sphere in the UV–vis range from 300 nm to 1000 nm. Absorption coefficients were obtained from UV–vis (transmission–reflection) and PDS (absorptance) data by measuring the layer thickness. In the energy range of strong absorption the absorption coefficient obtained from PDS is compared and scaled to transmission–reflection measurements which give a more accurate absolute value of the absorption coefficient in this regime. PDS, EQE and *J*–*V* measurements were done before any exposure to air had occurred. Samples for PDS were mounted in a cuvette containing the liquid FC75 inside the glovebox. Solar simulator and EQE measurements were done under inert atmosphere by mounting the samples (while still inside a glovebox) in a closed holder with glass window. Current–voltage curves were performed with an AM1.5 spectrum on a grade AAA Solar Simulator. No masks were used for solar simulator measurements since the *J*_sc_ values were obtained from EQE measurements. The reflection from the glass lid of the sample holder (8–10% depending on wavelength) was measured and accounted for during EQE analysis.

**Calculations for performance limitation:** The band gap is the only parameter needed to calculate the Shockley–Queisser limit of a certain material. The inflection point IP of the falling edge of EQE data served as value for the band gap [59]. The EQE IP obtained from measurements within this work was taken for all Sb_2_S_3_ data points. Other material’s IP were estimated from published EQE data. The FF is calculated by Green’s approximation [70] which was shown to be accurate in the regimes depicted in Figure 5a and 5b [73]. In Figure 5a the SQ limit of the *V*_oc_ and *n*_id_ = 1 is assumed. In Figure 5b the FF–*V*_oc_ relation is shown for the boundary cases of *n*_id_ = 1 and *n*_id_ = 2.

## Supporting Information

File 1 Additional Figures and Tables.
