# Supplementary material for: Spin-coated planar Sb2S3 hybrid solar cells approaching 5% efficiency
Source: Beilstein J Nanotechnol. 2018 Aug 8;9:2114–24. doi: 10.3762/bjnano.9.200 (PMC6122191; doi:10.3762/bjnano.9.200)
Supplement: File 1 — Additional Figures and Tables. [file Beilstein_J_Nanotechnol-09-2114-s001.pdf]

Supporting Information

for

Spin-coated planar Sb_2S_3 hybrid solar cells reaching 5% efficiency

Pascal Kaienburg*[1], Benjamin Klingebiel[1] and Thomas Kirchartz*[1,2]

Address: [1]IEK5-Photovoltaics, Forschungszentrum Jülich, 52425 Jülich, Germany and [2]Faculty of Engineering and CENIDE, University of Duisburg-Essen, Carl-Benz-Str. 199, 47057 Duisburg, Germany

Email: Pascal Kaienburg* - paskai@posteo.de, Thomas Kirchartz* - t.kirchartz@fz-juelich.de

* Corresponding author

Additional Figures and Tables

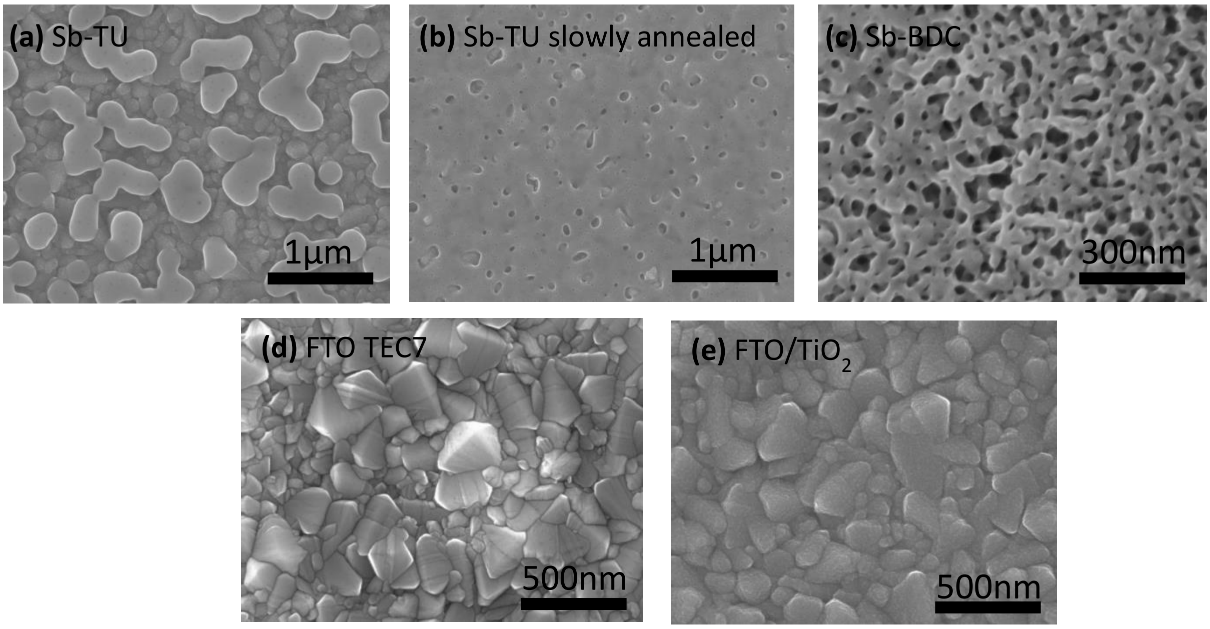

Figure S1: SEM images of amorphous samples (a–c) where the precursor is thermally decomposed but films are not yet crystallized. (c) looks like reported in [1]. For clarity bare FTO substrate (d) and TiO_2-coated FTO (e) is shown as well.

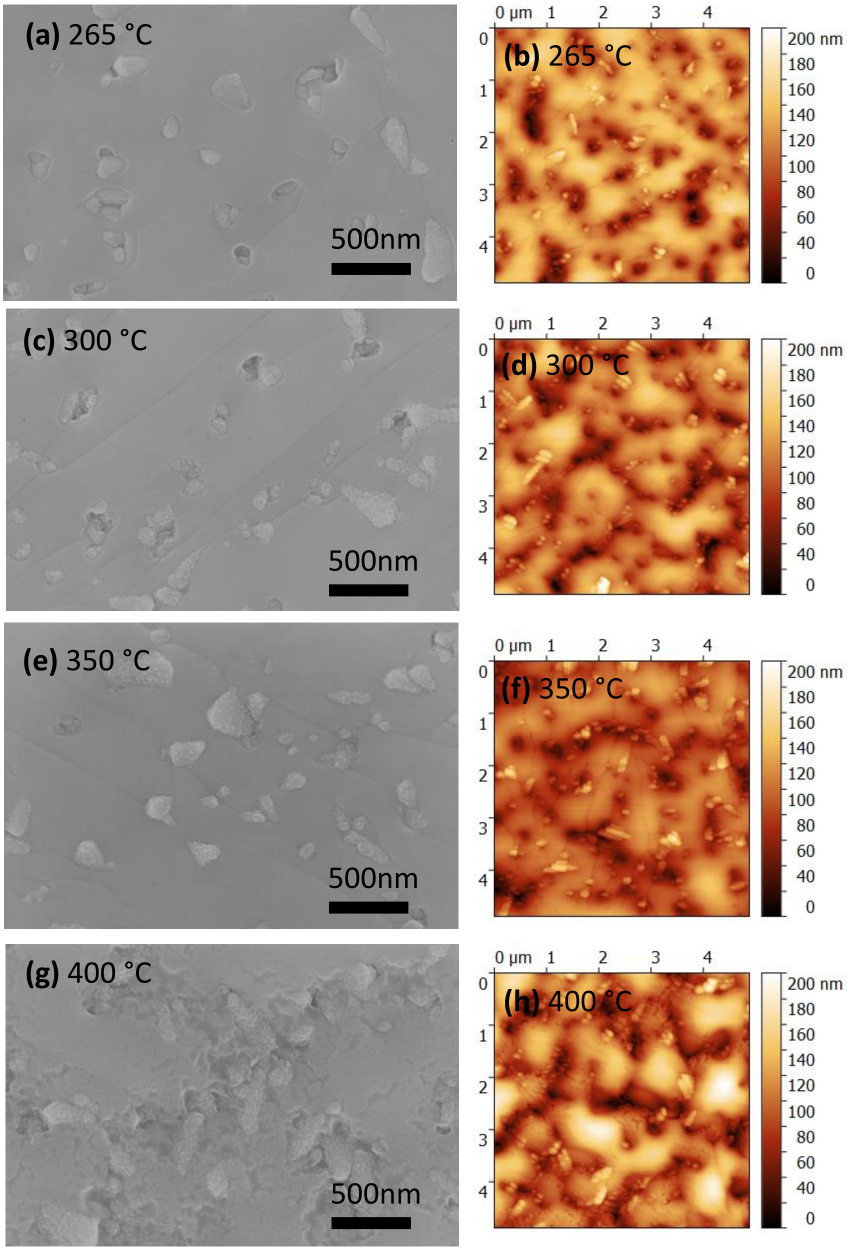

Figure S2: SEM (a,c,e,g) and AFM (b,d,f) images of Sb-TU samples crystallized at different temperatures. (a,b,d) are also shown in the main paper.

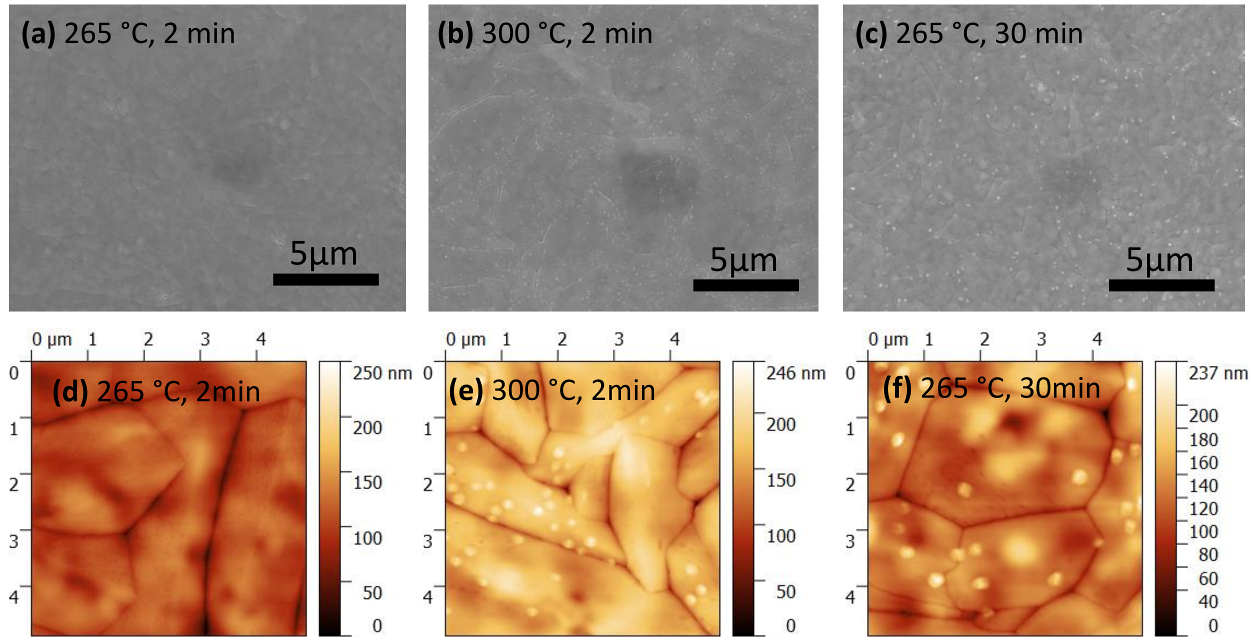

Figure S3: Zoomed-out SEM images (a–c) and AFM scans (d–f) of the Sb-BDC process. Grain sizes are similar through (a–c). The underlying FTO morphology is visible. Unlike in (c) and especially (b) no pyramidal features are observed in (a).

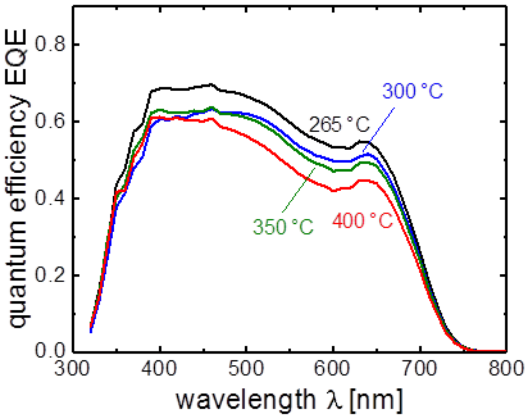

Figure S4: External quantum efficiency of the Sb-TU annealing study corresponding to the current–voltage curves shown in Figure 2d.

Table S1: Solar cell parameters of the Sb-TU annealing study corresponding to the current–voltage curves shown in Figure 2d.

T_{anneal} [°C]	V_{oc} [mV]	FF [%]	J_{sc} [mA cm^{-2}]	$J_{sc,EQE}$ [mA cm^{-2}]	PCE [%]	PCE_{corr} [%]
265	570	44.8	10.6	12.0	2.71	3.06
300	539	45.6	10.1	11.0	2.48	2.72
345	406	42.4	10.1	10.9	1.74	1.87
400	280	37.3	9.2	10.0	0.96	1.04

Table S2: Solar cell parameters of the Sb-BDC annealing study corresponding to the current–voltage curves shown in Figure 2h.

T_{anneal} [°C]	t_{anneal} [min]	V_{oc} [mV]	FF [%]	J_{sc} [mA cm^{-2}]	PCE [%]
265	2	605	46.1	14.1	3.94
300	2	567	41.0	13.0	3.02
265	30	563	43.7	13.0	3.19

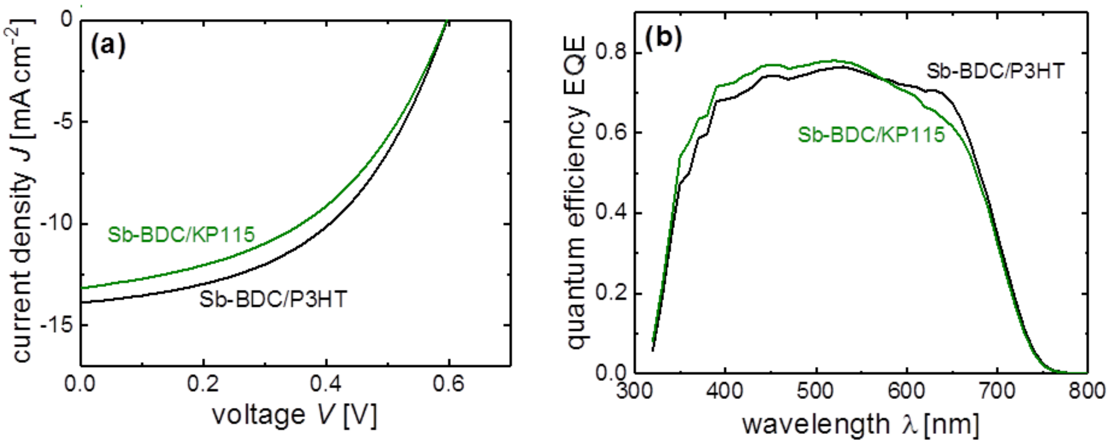

Figure S5: Comparison of the Sb-BDC process with KP115 as HTM shown in the main part with a P3HT device fabricated on the same day which did not reach the efficiency of the Sb_2S_3/P3HT cell presented in the main paper.

References

(1) Wang, X.; Li, J.; Liu, W.; Yang, S.; Zhu, C.; Chen, T. *Nanoscale* **2017**, *9*, 3386–3390. doi:10.1039/C7NR00154A
